# Investigating the relationship between prenatal androgen exposure and sleep quality: a comprehensive study using anthropometric measurements, questionnaires, actigraphy, and polysomnography

**DOI:** 10.3389/fendo.2024.1480963

**Published:** 2025-01-16

**Authors:** Adrian Bartoszek, Magdalena Sawic, Karol Pierzchała, Aleksandra Kudrycka, Piotr Białasiewicz, Wojciech Kuczyński

**Affiliations:** ^1^ Department of Bioanalytics, Medical University of Lublin, Lublin, Poland; ^2^ Independent Unit of Spectroscopy and Chemical Imaging, Medical University of Lublin, Lublin, Poland; ^3^ Department of Sleep Medicine and Metabolic Disorders, Medical University of Lodz, Lodz, Poland

**Keywords:** digit ratio, sleep, sleep quality, polysomnography, 2D:4D, PAE

## Abstract

**Background:**

The 2D:4D digit ratio (DR), representing the relative length of the index finger to the ring finger, is an anthropometric marker that shows sexual dimorphism, with males typically having a lower ratio than females. This parameter is linked to prenatal androgen exposure (PAE), which influences sexual differentiation of the brain and behavior. This study aimed to investigate the correlation between PAE and sleep among young adults.

**Methods:**

Anthropometric measurements were used to assess PAE, while sleep was evaluated using the Pittsburgh Sleep Quality Index (PSQI), Morningness-Eveningness Questionnaire (MEQ), actigraphy, and polysomnography (PSG). Data was collected from 720 participants via surveys, with 290 completing all questionnaires. Of these, 138 underwent anthropometric measurements, and 38 were further recruited for actigraphy, PSG.

**Results:**

Men exhibited a lower 2D:4D ratio than women, indicating higher PAE. The average PSQI score was 6.94 ± 2.98, and the MEQ score was 35.43 ± 19.59, with no significant gender differences in sleep quality or chronotype. Low PAE was associated with better sleep efficiency and a morning chronotype, but worse sleep quality in males. Actigraphy results showed no significant differences based on PAE. PSG data revealed that males with longer 2nd fingers and higher 2D:4D ratios were more likely to suffer from mild sleep apnea, a pattern not observed in women. PAE did not significantly impact other sleep architecture parameters.

**Conclusions:**

PAE, indicated by the 2D:4D ratio, is linked to sleep efficiency and chronotype, with significant gender differences. Men with lower PAE exhibited poorer sleep quality, and those with certain finger length patterns were more prone to mild sleep apnea. These findings underscore the potential long-term impacts of PAE on sleep health and emphasize the need for further research into gender-specific sleep regulation mechanisms.

## 1 Introduction

Sleep is a fundamental biological process vital for sustaining life and promoting overall health and well-being, impacting numerous physiological and psychological processes (1). Chronic sleep deprivation and poor sleep quality are linked to a wide range of health issues, including obesity (2), diabetes (2, 3), mental illnesses, hypertension (2, 4) cardiovascular disorders (5), and many others. Moreover, sleep deprivation can increase stress levels, impair cognitive and metabolic functions, and disrupt neuroendocrine processes, collectively impacting sexual and reproductive health (6). Sleep is vital for immune function, memory consolidation, and the clearance of brain metabolites (2). Despite its importance, the factors influencing sleep patterns are not fully understood. However, it is well known that men and women have different sleep patterns (7). Although the mechanisms behind sleep are understood, the reasons for these gender differences in sleep behavior remain unclear and underexplored. Women often experience lower sleep quality and increased disruptions throughout life (8). Pengo, Won, and Burjeily (2018) discuss how women face unique sleep challenges, including more frequent disruptions due to hormonal fluctuations across life stages such as menstruation, pregnancy, and menopause (9). Clinical and experimental studies suggest that sex steroids significantly influence sleep regulation (10). Investigating the correlation between neuroendocrine factors and sex differences is critical for understanding sleep-related disorders. While research into how sex differences and sex steroids modulate sleep is progressing, it remains in an early stage, highlighting the need for further exploration into these complex interactions to improve therapeutic approaches (11).

One of the biomarkers of prenatal sex steroids, first highlighted by Manning et al. (1998) is the relative ratio of the second to fourth finger (2D:4D) (7). This ratio is typically lower in males, reflecting a longer ring finger compared to the index finger, whereas females exhibit a more similar length between these digits (12). The 2D:4D (also known as digit ratio DR) is widely utilized as a noninvasive marker of prenatal androgen exposure (PAE) and serves as a valuable biomarker for various conditions (13). Sex differences in 2D:4D ratios are established early in life and remain stable, unaffected by pubertal growth or hormonal changes. This consistency reflects the influence of PAE, making the DR a reliable marker for studying prenatal hormone effects on sex-specific traits (14). However, a recent review conducted by Manning and Fink (2023) elaborates on underscores the utility of 2D:4D as a biomarker influenced by prenatal testosterone and estrogen, with supporting evidence from diverse non-human tetrapods. Despite its sexual dimorphism in humans, the moderate effect size raises questions about its precision as a proxy for fetal hormones. Variability in fetal exposure to a “sex-steroid cocktail” from gonadal and maternal sources may account for these discrepancies. The proposed methodological refinements, including precise hormone sampling and digit measurements during the critical first trimester, aim to clarify 2D:4D’s biological significance in human developmental research (15). Anderssen et al. emphasized that understanding the relationship between sleep and testosterone extends beyond theoretical interest, as it may illuminate mechanisms underlying health issues related to sleep disturbances, aging, and shift work (16). Consequently, alterations in hormonal levels, including testosterone, are to be anticipated (16). Verster et al. (17) found no significant correlation between DR and sleep quality in females; however, a positive correlation between right DR and total sleep time was observed in males. This study had several limitations, such as the lack of quantitative sleep measurements in their analysis.

The simultaneous investigation of 2D:4D and sleep quality in academic research is underpinned by their shared connection to PAE, a critical factor influencing early developmental pathways. The 2D:4D ratio acts as a non-invasive biomarker for androgenic influence during gestation, while sleep quality is closely regulated by neuroendocrine systems sensitive to hormonal fluctuations. We believe that correlating these traits together allows us to investigate the interplay between early hormonal environments and sleep-related outcomes, potentially shedding light on long-term health implications influenced by prenatal androgens. This study aims to explore the relationship between PAE, as indicated by the DR (2D:4D), and various sleep parameters, providing new insights into how prenatal hormonal exposure may impact sleep and overall well-being.

## 2 Materials and methods

### 2.1 Study design

This study is the part of the research project: “Understanding the associations of prenatal androgen exposure on sleep physiology, circadian proteins, anthropometric parameters, hormonal factors, quality of life and sex among healthy young adults – BOAT international, multicenter study” (18).

Surveys were conducted from March 2020 to March 2022. The inclusion and exclusion criteria, along with the research protocol, have been detailed in previous publications (18). Data was collected from 720 participants, with 290 of them completing all questionnaires. Subsequently, 138 individuals underwent anthropometric measurements, and finally, 39 were recruited for Actigraphy, polysomnography (PSG).

### 2.2 Measurement tools

#### 2.2.1 Phase 1: Questionaries and anthropometric measurements

Participants completed questionnaires, including:

- Demographic: Collected information on age, ethnicity, sex, gender, hand dominance, medical conditions, previous hand injury.
- Pittsburgh Sleep Quality Index (PSQI): Assessed sleep quality and disturbances over one month, evaluating seven domains: sleep latency, duration, efficiency, disturbances, medication use, daytime dysfunction, and overall sleep quality. This yields a comprehensive overview of an individual’s sleep patterns (19).
- Morningness-Eveningness Questionnaire (MEQ): determined circadian preference based on self-reported optimal sleep and activity times. Individuals are categorized as either a “morning type,” “evening type,” or “neither” based on their self-reports (20).

The direct measurements of the second and fourth finger on both hands were performed by qualified staff using a sliding calliper (Vernier calliper) with an accuracy of 0.001 m. Based on values of the fingers length, the 2D:4D index was calculated as a quotient of the length of the second digit and the fourth digit (mm). Measurements were performed on the palmar side of the hand using anthropometric points lying on the digit axis: pseudophalangion—a point in the finger metacarpophalangeal crease, dactylion—the most distal point on the fingertip. 2D:4D ratio > 1 was considered as a low PAE, while ratio <1 as high PAE.

#### 2.2.2 Phase 2: Actigraphy and polysomnography

- Actigraphy: Used to evaluate sleep-wake parameters objectively. This noninvasive wristwatch-like device measures rest-activity data based on movements, providing information on daily and nocturnal activity, average sleep time, sleep continuity, awakenings, and naps (21).
- PSG: The gold standard for sleep examinations (22), distinguishing and examining every sleep phase. It uses EEG, electrooculogram, and electromyogram to present three states: vigilance, nREM, and REM sleep.

Each participant was asked for a 7 days quantitative daily and nocturnal activity recording using portable actigraphy. On the last day, one-night PSG data (total sleep time, duration of REM and nREM phases, apnea-hypopnea index, arousal index, and saturation) was captured with a Nox A1 (ResMed).

### 2.3 Data collection

All participants were aware of the study conditions and gave informed consent to participate. Confidentiality and anonymity were maintained and no data that could help identify a responder were collected. The study is conducted in accordance with the amended Declaration of Helsinki, and the Ethics Committee of the Medical University of Lodz approved the study protocol (RNN/394/19/KE); Miniatura 4, National Science Centre—no 2020/04/X/NZ4/00564.

### 2.4 Statistical analyses

The data collected were verified for completeness, quality, and consistency. Statistical analysis was performed using Stata (version 18.0). A level of 5% was used as a significance threshold for all the results unless stated otherwise. In the case of multiple testing, the Bonferroni correction was used. No data obtained had normal distribution (Shapiro–Wilk’s test, p < 0.05). The relationship between the independent subgroups was assessed using the Mann-Whitney U test. The relationship between continuous variables was assessed using Spearman correlation coefficient.

## 3 Results

### 3.1 Study group

The study included Polish students with an average age of 22.28 ± 1.99 years with no medical conditions and no previous hand-injury. Among them, 61.59% were women. The BMI distribution shows that 76.81% of individuals are of normal weight, 10.87% overweight, 10.14% underweight, and 2.17% are obese (mean BMI 21.95 ± 1.99). Individuals recruited for sleep assessments are 76,32% of normal weight, 13,16% underweight and 13.53 overweight. 94.2% of the respondents were right-handed. Anthropometric measurements and PAE considering the sex of the respondents are presented in **Tables 1** and **2**. Men have larger 2nd and 4th fingers, while women have a significantly larger 2D:4D ratio (**Tables 1**, **2**).

**Table 1:** Anthropometric measurements.

Anthropometric measurements	Mean	Gender	
		Female (61.59%)	Male (38.41%)
2D _L_	70.616 (4.717)	**68.494 (3.984)**	**74.019 (3.718)**
4D _L_	71.043 (4.559)	**69.129 (4.217)**	**74.113 (3.232)**
2D:4D _L_	71.043 (4.551)	**69.118 (4.193)**	**74.132 (3.223)**
2D _R_	71.086 (5.192)	**68.776 (4.457)**	**74.792 (4.026)**
4D _R_	71.333 (5.087)	**68.988 (4.492)**	**75.094 (3.488)**
2D:4D _R_	71.304 (5.071)	**68.988 (4.481)**	**75.019 (3.533)**
2D:4D _mean_	0.994 (0.032)	0.997 (0.033)	0.990 (0.029)
2D:4D *_handedness_*	0.997(0.033)	**1.03 (0.033)**	**0.988 (0.032)**

In bold-statistically significant differences. _L, left hand measurement; _R, right hand measurement; _handedness, dominant hand measurement. Data presented as mean (SD).

**Table 2:** PAE and Gender.

PAE		%	Gender		p
			Female (%)	Male (%)	
L	high	50.7	29.7	21.0	0.45
	low	49.3	31.9	17.4	
R	high	51.5	27.5	23.9	**0.045**
	low	48.5	34.1	14.5	
mean	high	54.35	29.7	24.6	0.06
	low	46.65	31.9	13.8	
handedness	high	50.0	26.8	23.2	0.05
	low	50.0	34.8	15.2	

In bold-statistically significant differences. _L, left hand measurement; _R, right hand measurement; _handedness, dominant hand measurement. Data presented as mean (SD).

The average score for the PSQI is 6.94 ± 2.98, and for the MEQ it is 35.43 ± 19.59. No significant differences were observed in sleep quality or chronotype between genders. However, at the borderline of statistical significance, men were more likely to use sleep medications.

### 3.2 Relationship between PAE, sleep and chronotype

Participants with low PAE (measured on dominant hand), had significantly better sleep efficiency according to PSQI and had more morning chronotype (**Table 3A**). In males, low PAE was associated with worse sleep quality. No changes in sleep or chronotype were observed among women (**Table 3A**). In females, 2D:4D was negatively associated with sleep quality, but the correlation was weak (**Table 3B**).

**Table 3A:** PSQI, MEQ and PAE.

	PAE *_handedness_*		PAE *_handedness_*			
			Male		Female	
	high (50.00%)	Low (50.00%)	High (43.53%)	Low (56.47%)	High (60.38%)	Low (39.62%)
1. Sleep Quality	0.81 (0.71)	1.0 (0.84)	**0.68** **(0.67)**	**1.06** **(0.84)**	0.97 (0.74)	0.86 (0.85)
2. Sleep Latency	1.22 (1.06)	1.14 (0.96)	1.22 (1.13)	1.1 (0.97)	1.22 (0.97)	1.24 (0.94)
3. Sleep Duration	1.39 (1.20)	1.03 (1.06)	1.49 (1.15)	1.06 (1.12)	1.28 (1.28)	0.95 (0.92)
4. Sleep Efficiency	**1.71** **(1.13)**	**1.32** **(1.16)**	1.78 (1.06)	1.35 (1.16)	1.62 (1.21)	1.24 (1.18)
5. Sleep Disturbance	0.86 (0.65)	0.87 (0.51)	0.86 (0.67)	0.85 (0.55)	0.84 (0.63)	0.9 (0.44)
6. Sleep Medication	0.19 (0.65)	0.17 (0.59)	0.27 (0.84)	0.25 (0.7)	0.09 (0.3)	0.0 (0.0)
7. Sleep Dysfunction	1.1 (0.99)	1.07 (0.88)	1.19 (1.05)	1.02 (0.84)	1.0 (0.92)	1.19 (0.98)
PSQI	7.35 (3.14)	6.54 (2.42)	7.65 (3.51)	6.54 (2.35)	7.0 (2.65)	6.52 (2.64)
MEQ	**31.52** **(19.89)**	**39.35** **(18.62)**	30.62 (19.84)	38.33 (20.79)	32.56 (20.21)	41.67 (12.46)

In bold-statistically significant differences. _handedness, dominant hand measurement. Data presented as mean (SD).

**Table 3B:** PSQI, MEQ and 2D:4D.

	2D:4D *_handedness_*	2D:4D *_handedness_*	
		Male	Female
1. Sleep Quality	-0.04	0.25	**-0.15**
2. Sleep Latency	-0.13	0.04	-0.05
3. Sleep Duration	0.05	-0.10	-0.13
4. Sleep Efficiency	-0.31	-0.10	-0.21
5. Sleep Disturbance	-0.24	0.01	-0.03
6. Sleep Medication	-0.02	-0.06	-0.14
7. Sleep Dysfunction	-0.05	-0.11	0.06
PSQI	-0.19	-0.09	-0.17
MEQ	0.06	0.15	0.20

In bold-statistically significant coefficient correlation. _handedness, dominant hand measurement.

### 3.3 Relationship between PAE and actigraphy

Participants were asked to wear portable actigraphy for 7 consecutive days to assess rest and activity cycles. PAE (measured on dominant hand) did not differentiate respondents in terms of parameters assessed by actigraphy (**Table 4**).

**Table 4:** Actigraphy among participants concerning PAE.

Actigraphy	PAE *_handedness_*		PAE *_handedness_*			
			Male		Female	
	high (52.78%)	low (47.22%)	high (70.59%)	low (29.41%)	high (36.84%)	low (63.16%)
Sleep Efficiency (%)	73.83 (6.92)	71.55 (7.42)	72.71 (7.03)	74.52 (2.92)	75.75 (6.82)	70.31 (8.45)
Number of Active Periods	74.55 (45.75)	71.09 (32.84)	76.1 (42.73)	57.97 (22.18)	71.9 (54.02)	76.56 (35.76)
Activity Length (min)	97.23 (18.99)	101.02 (15.24)	94.93 (12.57)	104.47 (17.44)	101.17 (27.63)	99.58 (14.82)
Total Elapsed time (min)	540.47 (83.84)	512.59 (83.34)	536.0 (85.38)	474.8 (43.79)	548.14 (87.25)	528.33 (92.12)
Total Sleep Time (min)	383.37 (47.73)	363.12 (66.15)	370.58 (50.43)	348.8 (11.39)	405.29 (35.83)	369.08 (78.65)
Total Wake Time (min)	156.11 (71.27)	148.53 (55.21)	164.33 (68.22)	125.4 (36.91)	142.0 (79.61)	158.17 (59.95)

_handedness - dominant hand measurement. Data presented as mean (SD).

### 3.4 Relationship between PAE and PSG

On the last day of actigraphy monitoring participants were tested with PSG. In our sample, 22.22% suffered from mild sleep apnea according to apnea–hypopnea index (AHI) and no respondents suffered from moderate or severe apnea. Respondents with longer right or left 2nd finger more often suffered from mild sleep apnea (**Table 5A**). Males with longer right or left 2nd finger and higher 2D:4D (measured on left hand) more often suffered from mild sleep apnea (**Table 5A**). The length of the fingers did not differentiate sleep apnea in women.

**Table 5A:** Anthropometric measurements and AHI.

	Mild (22.22%)	Normal (77.78%)	Male		Female	
			Mild (35.29%)	Normal (64.71%)	Mild (10.53%)	Normal (89.47%)
2D _L_	**73.75** **(5.39)**	**69.54** **(4.05)**	**76.33** **(2.94)**	**72.73** **(2.76)**	66.0 (0.0)	67.47 (3.37)
4D _L_	73.75 (4.3)	69.89 (5.25)	75.83 (2.23)	74.36 (2.98)	67.5 (0.71)	67.0 (4.29)
2D:4D _L_	1.0 (0.02)	1.0 (0.04)	**1.01** **(0.02)**	**0.98** **(0.03)**	0.98 (0.01)	1.01 (0.04)
2D _R_	**74.12** **(4.49)**	**70.39** **(4.11)**	**76.33** **(2.16)**	**73.45** **(2.84)**	67.5 (0.71)	68.41 (3.59)
4D _R_	73.75 (4.43)	70.43 (5.44)	76.0 (1.67)	75.18 (2.82)	67.0 (1.41)	67.35 (4.4)
2D:4D _R_	1.01 (0.03)	1.0 (0.04)	1.0 (0.03)	0.98 (0.03)	1.01 (0.03)	1.02 (0.04)
2D:4D _mean_	1.0 (0.02)	1.0 (0.04)	**1.01** **(0.02)**	**0.98** **(0.02)**	0.99 (0.02)	1.01 (0.04)
2D:4D *_handedness_*	1.01 (0.03)	1.0 (0.04)	1.0 (0.03)	0.98 (0.03)	1.01 (0.03)	1.02 (0.04)

In bold-statistically significant differences. _handedness - dominant hand measurement. Data presented as mean (SD).

PAE did not differentiae respondents in terms of sleep architecture assessed by PSG (**Table 5B**).

**Table 5B:** PSG among participants concerning PAE.

PSG	PAE *_handedness_*		PAE *_handedness_*			
			Male		Female	
	high (52.78%)	low (47.22%)	high (64.71%)	low (35.29%)	high (36.84%)	low (63.16%)
TST (min)	351.78 (60.25)	353.03 (73.87)	332.64 (55.77)	352.83 (48.82)	381.86 (58.09)	353.12 (85.74)
SL (min)	32.18 (25.48)	46.46 (35.61)	29.07 (21.89)	46.08 (35.45)	37.07 (31.55)	46.65 (37.26)
N1%TST	1.96 (1.28)	1.42 (0.83)	1.81 (1.16)	1.58 (1.11)	2.2 (1.51)	1.34 (0.69)
N2%TST	55.26 (8.31)	59.94 (10.29)	56.77 (9.07)	57.75 (9.04)	52.87 (6.91)	61.04 (11.08)
N3%TST	23.19 (7.05)	19.51 (6.81)	22.61 (6.87)	20.52 (5.43)	24.1 (7.79)	19.01 (7.59)
REM%TST	19.59 (4.84)	17.98 (8.07)	18.8 (5.15)	20.13 (5.54)	20.83 (4.39)	16.91 (9.1)

_handedness - dominant hand measurement; TST-Total Sleep Time; SL-Sleep Latency; N1,2,3% Percentage of time spent in Stage N1,N2,N3 (Non-REM Stages); REM% -Percentage of time spent in REM. Data presented as mean (SD).

## 4 Discussion

Despite the critical role of sleep in maintaining health and daily functioning, approximately 70 million individuals in the United States and nearly 45 million in Europe suffer from chronic sleep disorders and sleep deprivation (23). These conditions significantly impair quality of life by disrupting cognitive performance, emotional well-being, and physical health (24). Addressing this widespread health issue affecting millions of individuals, we conducted a study aimed to investigate the potential correlation between PAE, as indicated by the 2D:4D ratio. To gain a comprehensive understanding of the subject, we used a variety of assessments. These included the PSQI and the MEQ, as well as objective sleep evaluations using actigraphy and PSG. These tools allowed us to obtain a detailed and accurate picture of the participants’ sleep patterns. To date, to the best of our knowledge, only a few papers follow the relationship between sleep, PAE, and DR and these papers rely mainly on questionnaires.

### 4.1 Gender differences in 2D:4D and sleep quality

Firstly, we compared sex dimorphism in 2D:4D ratios within our cohort if it aligns with existing literature (12). Males exhibited lower 2D:4D ratios (**Table 1**) than females, which is consistent with the hypothesis that higher prenatal androgen levels result in lower 2D:4D ratios. Similar findings were observed by Mackus et al., where gender-specific links between the 2D:4D digit ratio, with these associations being more pronounced in men than in women (25). However, the lack of gender-based differences in general sleep quality diverges from studies suggesting poorer sleep among females (26), underscoring the need for further nuanced investigations. In males, lower PAE was linked to reduced sleep quality, potentially reflecting androgenic influences on circadian regulation and stress responsiveness through the hypothalamic-pituitary-adrenal axis. Contrastingly, females showed weak associations, suggesting gender-modulated pathways. Studies like Vester et al. (2017) support these differential effects, indicating sex hormones’ varied roles in shaping sleep physiology (17).

Another interesting finding was made by Srihari et al. (2014) (27). Their study aimed to investigate the correlation between DR and obstructive sleep apnea (OSA). Although it is not directly similar to our study, the authors investigated the risk for OSA with a modified Berlin questionnaire and evaluated excessive daytime sleepiness using the Epworth Sleepiness Scale. Similar to our findings, they noticed gender differences in the 2D:4D ratio (27). Contrary to our study they reported gender differences in mean 2D:4D, but we only found significant variation taken on the dominant hand. Regarding sleep quality, the study categorized participants based on their snoring habits and excessive daytime sleepiness, but it did not explicitly detail gender differences in sleep quality outcomes. This study also differs from ours in the way the fingers were measured. Our study used a sliding calliper (Vernier calliper) with an accuracy of 0.001. In the cited study Digital photographs of the ventral surface of both hands were taken. The lengths of the 2D and 4D were measured from the bottom-most crease to the tip of the finger pad using an online ruler. This may have significantly affected the 2D2:4D values as indicated in the literature (27). A meta-analysis of studies on the 2D:4D ratio identified variability based on these techniques, with direct measurements often yielding lower ratios compared to photocopies or scans, emphasizing the need for methodological consistency in comparative research (29, 30). As was mentioned earlier, our DR results differ marginally from Srihari et al. (2014) (27). This may be influenced by the study group characteristics, in their case it was more diverse in age (20-45 compared to ours 22.28 ± 1.99. Moreover, a significant gender disparity was observed in their study- 77% men and only 23% women compared to 62% for female and 38% for male.

Additionally, another interesting finding was made by Kalichman et al. (2017) (31). They categorized individuals based on their 2D:4D into different types or groups. Specifically, they identified three types of finger length patterns: Type 1: Characterized by a lower 2D:4D ratio. Type 2: Intermediate 2D:4D ratio. Type 3: Higher 2D:4D ratio. In our study, we used a similar methodology, with grouping: low and high PAE. The analysis revealed that individuals with a higher 2D:4D ratio (Type 3) exhibited significantly higher osseographic scores. Moreover, as in our study, males tend to have a lower 2D:4D ratio than females. The authors used indirect measurement methods in the study. Single plain radiographs of both hands were taken standardized, ensuring consistent exposure and positioning to avoid variations in film development. Each hand was visually classified based on the relative lengths of 2D and 4D (31). However, Butovskaya et al. (2023) found a significant correspondence between radiographic images and direct measurements of 2D:4D ratios (32). Specifically, the correlation between left-hand 2D:4D ratios obtained from radiographs and those measured directly from soft tissue was approximately r ~ 0.60. This suggests that the DRs measured from soft tissue correspond well with those derived from radiographic images, indicating that both methods yield comparable results in assessing 2D:4D ratios. However, the study has several limitations, following methodological tradition and the fact that radiographic data were obtained for the left hand only. Moreover, there were challenges in accurately measuring soft-tissue fingertips from radiographic images, leading to potential inconsistencies in the data. This limits the opportunity to compare their results with those of several previous studies (32).

### 4.2 Sleep quality and chronotype

The chronotype categorizes individuals as morning or evening types, morning-oriented individuals often align better with societal norms and report improved mental health outcomes, while evening types may face challenges such as circadian misalignment and increased susceptibility to mood disorders (33, 34). In our study, individuals with high PAE tended to have a morning chronotype, indicating a potential influence of prenatal androgens on circadian preferences. Moreover, no changes in sleep or chronotype were observed among women (**Table 3A**). A significant relationship was observed between high PAE (lower 2D:4D ratio) and poorer sleep efficiency as measured by the PSQI, particularly among males. This finding suggests that higher prenatal androgen levels may negatively impact sleep efficiency in adulthood. Interestingly, while men with lower PAE (higher 2D:4D ratio) exhibited worse sleep quality, this pattern was not observed in women. This gender-specific effect might reflect the differential impacts of prenatal androgens on sleep regulation mechanisms between males and females. In females, although there was a negative association between 2D:4D and sleep quality, the correlation was weak, indicating that other factors may play a more prominent role in determining sleep quality in women. Additionally, Muzni et al. (2020) suggested that in young adults (as our study group), self-reported sleep quality is more strongly linked to mental and physical health than factors such as chronotype and sleep duration (35). Our group contained mainly medical university students, which may have contributed significantly to the results described above due to their high risk maintaining mental health issues correlated with studies (36).

### 4.3 Objective sleep measures: actigraphy and PSG

There are just a few studies on DR in which PSG was used for sleep efficiency evaluation. Goel et al. (2005) found out that young, healthy women has better sleep quality than young, healthy men, which was not observed in our study (37). Different outcomes may arise from the fact that PSG has its limitations, such as patient comfort. There are also technical issues: the complexity of PSG setups can lead to technical failures, such as electrode malfunctions or data recording errors, which may compromise the integrity of the result (28).

The present study did not identify any significant differences in sleep architecture, as assessed by PSG (**Table 5B**), or in the parameters assessed by actigraphy, between the high and low PAE respondents. Verster et al. conducted a similar study in 2017 (17). This study aimed to explore the potential correlation between the 2D:4D ratio and various sleep parameters, including insomnia, sleep quality, and total sleep duration. The survey was conducted on Dutch students. Data from 143 men and 366 women were analyzed, with a mean (SD) age of 20.8 (2.6) years, similar to our study group. Overall, no significant correlations were found between the DR of either hand and insomnia, sleep quality, or total sleep time. However, in men, a significant correlation was observed between the right 2D:4D ratio and total sleep time (r=0.173, p=0.039). No significant effects were noted in women, similar to our study results. To the best of our knowledge, our study represents the first attempt to correlate DR with actinography. However, our results did not yield any statistically significant findings (**Table 4**).

## 5 Implications and future research

These findings suggest that PAE, as indicated by the 2D:4D ratio, has nuanced effects on sleep quality and certain sleep disorders, with notable gender differences. The lack of significant findings in some areas highlights the complexity of sleep regulation and the need for further research to elucidate the mechanisms underlying these associations.

Future studies should consider larger sample sizes and longitudinal designs to better capture the developmental trajectories influenced by PAE. Additionally, investigating the interplay between PAE, sleep, and other physiological and psychological factors could provide deeper insights into the long-term impacts of prenatal hormone exposure.

## 6 Limitations

The study sample consisted predominantly of young, healthy adults, which may limit the generalizability of the findings to other age groups or individuals with concurrent health conditions. While the study accounted for several variables, other potential confounding factors, such as stress, dietary habits, and environmental influences, were not controlled for, which could affect sleep quality. The cross-sectional nature of the study limits our ability to draw causal inferences. Longitudinal studies would be invaluable in establishing temporal relationships between PAE and sleep outcomes. Additionally, our study group for objective sleep measures was relatively small, and future research with larger cohorts would help validate our findings. Furthermore, the reliance on self-reported data for some measures could introduce bias, despite our efforts to corroborate these findings with objective data. Data collection was also impacted by the COVID-19 pandemic, which may have introduced variability in the study conditions and affected the overall participation rate.

### 6.1 Strengths

The study exhibits several strengths that enhance the reliability and validity of its findings. The use of both subjective (PSQI, MEQ) and objective measures to assess sleep (actigraphy and PSG) - provides a comprehensive evaluation of sleep patterns. Additionally, the inclusion of anthropometric measurements, adds depth to the data, allowing for more nuanced insights into the relationship between PAE and sleep. The study’s robust methodology, including the use of validated questionnaires and advanced sleep monitoring techniques, ensures the accuracy and reliability of the results. Furthermore, the research addresses a significant gap in the literature by exploring the relationship between PAE and sleep, providing valuable contributions to the understanding of how prenatal factors influence adult sleep patterns and overall health. We believe that the advantage of our study is its high reliability of the methods used (eg digit measurements by calliper) and therefore brings to the study of the 2D:4D, which, as the literature indicates, is a good indicator of both diseases, anthropometric measurements, and many others.

## 7 Conclusions

This study contributes to the understanding of how PAE might influence sleep patterns and disorders in young adults. Contrary to the subjective sleep measures, PAE did not significantly differentiate participants in terms of actigraphy parameters, suggesting that daily rest-activity cycles might be less influenced by prenatal androgen levels. In terms of PSG measures, a notable finding was the association between finger lengths and mild sleep apnea, particularly among males. Men with longer 2nd fingers and higher 2D:4D ratios were more likely to suffer from mild sleep apnea. This association was not observed in women, highlighting a potential sex-specific vulnerability to sleep-disordered breathing influenced by PAE. However, PAE did not affect other aspects of sleep architecture, suggesting that its influence might be more specific to certain sleep disorders rather than overall sleep structure. The observed gender differences underscore the need for sex-specific analyses in future research. While PAE appears to affect sleep quality and apnea in males, its impact on other sleep parameters remains less clear, necessitating further investigation into the intricate relationships between early hormonal influences and adult sleep health.

## Data Availability

The raw data supporting the conclusions of this article will be made available by the authors, without undue reservation.
